# Reliability, Validity, and Responsiveness of the Chinese Learning Accomplishment Profile (C-LAP)

**DOI:** 10.3390/children8110974

**Published:** 2021-10-28

**Authors:** Deng Chen, Sikun Chen, Yilu Huang, Jinming Yu

**Affiliations:** Institute of Clinical Epidemiology, Key Laboratory of Public Health Safety, Ministry of Education, Key Lab of Health Technology Assessment of Ministry of Health, School of Public Health, Fudan University, Shanghai 200433, China; 19111020021@fudan.edu.cn (D.C.); skchen20@fudan.edu.cn (S.C.); 20211020036@fudan.edu.cn (Y.H.)

**Keywords:** child’s development, early childhood, reliability, validity, responsiveness

## Abstract

Objectives: To evaluate the reliability, validity, and responsiveness of the Chinese Learning Accomplishment Profile in China. Methods: 12,098 participants aged from 0 to 36 months from 30 provinces (mostly from Shanghai) in China were enrolled between 2013 and 2020. The reliability was reflected by Pearson correlation coefficients, Cronbach’s alpha coefficients and standard errors; the validity was shown by the coefficients between the dimensions, and we also evaluated the responsiveness as a supplement to the validity. Results: Reliability: in six domains among each subgroup, Pearson correlation coefficients between developmental age and chronological age ranged from 0.89 to 0.98, Cronbach’s alpha coefficients from 0.71 to 0.99, and standard errors from 0.15 to 2.76. Validity: after controlling for chronological age, the correlation coefficients between the dimensions were between 0.18 and 0.78, and most of them were below 0.70. Responsiveness: developmental age of all domains obtained via the Chinese Learning Accomplishment Profile system changed significantly (*p* < 0.001) with time (gap of 1–3 months), and the standardized response mean ranged from 0.66 to 2.45. Conclusions: The Chinese Learning Accomplishment Profile is suitable for assessing children’s development in Shanghai, but still needs confirmation when used in other provinces in China due to the great differences between regions in China.

## 1. Introduction

Children’s learning performance is highly associated with early neuropsychology development, especially when children are aged 0 to 3 years old [1]. In recent decades, many scales have emerged regarding children’s neuropsychological development, which can be divided into two types: normative assessment and criterion-referenced assessment. The former mainly evaluates the performance of a child by comparing it with that of normal children of his chronological age [2]. Despite obtaining the relative developmental level of the child, it is difficult for this type of assessment to provide teachers or parents with specific parenting guidance [3]. The tools of the criterion-referenced assessment provide a detailed sequential order of developmental skills in any one area of development [4,5], in which the items are skills, arranged from the easiest to the most complex. Only when the previous items are passed will the subsequent items be tested. These items, sorted by difficulty, can show in specific skills that children lack in different dimensions in more detail.

The Early Learning Accomplishment Profile (E-LAP), developed in 1969, is a classic criterion-referenced assessment system, the items of which were drawn from a number of well-known normative assessment tools [3]. After incorporating these items in sequential order, the E-LAP offers distinct advantages over other, more normative assessment devices, which were found to be reliable and valid [6]. The E-LAP examines six domains of child development including Gross Motor, Fine Motor, Cognitive, Language, Self-Help, and Social Emotional. Each domain can be used to assess children’s developmental age in different aspects. Although the E-LAP was developed to only be used in conjunction with other instruments to assess child development, and cannot be used as a diagnostic tool [6], it has been used separately in many studies to evaluate children aged from 0 to 36 months [7,8,9].

To better assist Chinese pediatricians with evaluating children’s development, we translated the E-LAP into a Chinese edition (Chinese Learning Accomplishment Profile (C-LAP)) with reference to the ITC guidelines for translating and adapting tests (ITC, 2017) [10].

The translation and adaptation processes considered linguistic, psychological, and cultural differences between China and USA through the choice of experts with relevant expertise. We selected samples of sufficient size and relevance for the empirical analyses, with relevant characteristics for the intended use of the test.

In this paper, the reliability and validity, and responsiveness were estimated to provide relevant statistical evidence of the adapted version of the assessment system in China.

## 2. Materials and Methods

### 2.1. Participants

A prospective study was conducted among 12,098 participants aged from 0 to 36 months from 30 provinces (mostly are from Shanghai) in China, between 2013 and 2020 (participants with abnormal results in the routine physical examination or diagnosed development disorders or diseases including cerebral palsy (CP), autism spectrum disorder (ASD), Down Syndrome (DS), etc., were excluded). Each child that participated in the study performed the C-LAP test to assess the developmental age, which refers to the extent of the child’s development at a certain age. Other information, such as parents’ educational level, age, and child’s chronological age, were obtained by standardized questionnaires for further study.

Written informed consent to participate was obtained from the parent or legal guardian of the child. This study was approved by the Institutional Review Board of the School of Public Health, Fudan University (IRB00002408, FWA00002399; approval number IRB#2019-04-0741).

### 2.2. Statistical Analysis

Due to the long period of data collection, the data in the research were separated into two groups, including data collected from 2013 to 2015, and data collected from 2016 to 2019, for analysis. Since most participants were from Shanghai, they were also divided into two groups, based on whether they were from Shanghai, or not for analysis. R 3.6.2 was adopted for statistical analysis [11]. All properties were two-sided, while only *p* < 0.05 was statistically significant.

### 2.3. Reliability

Reliability refers to the test’s consistency in replications [12]. Reliability can be evaluated with several methods, including the test-retest method, alternative-form method, split-half method, and inter-item consistency (internal consistency) method. In our research, we measured the correlations between chronological age and developmental age with Pearson correlation coefficients (r) and estimated Cronbach’s alpha, which infers the internal consistency. Internal consistency reflects the coherence of items in each domain. If the test’s Cronbach’s alpha is high enough, internal consistency would be excellent, which presents good reliability for this study. We also evaluated the standard error of measurement (*SEM*) of the C-LAP system. *SEM* is calculated by the following equation [13]:

$$SEM=S*\sqrt{1-\alpha }$$

where:

*SEM* = Standard error of measurement

*S* = Standard deviation of the test results

*A* = Cronbach’s alpha

*SEM* is negatively associated with the test’s reliability. If the test were completely reliable, the alpha coefficients would be 1, making *SEM* equal to 0. However, if the test were completely unreliable, with alpha coefficients of 0, *SEM* would be equal to the standard deviation of the test results.

### 2.4. Validity

Validity refers to the extent of the association between the test’s result and true characteristics, which is also considered to be the accuracy of the test [14]. Construct validity was obtained to represent the C-LAP’s validity. Pearson’s *r* coefficients were calculated between different domains to examine the relationship between the domains. With age considered to be an important confounder, we calculated the coefficients without and with age-adjustments via Pearson zero-order correlations and partial correlations.

### 2.5. Responsiveness

Responsiveness refers to the test’s ability to measure the clinical change [15], which is an aspect of validity. We selected participants who had completed the C-LAP test twice. These subjects were divided into three groups according to the time gap between the two tests for analysis. Paired *t*-tests were used to observe whether the subjects showed any changes between the two tests. We calculated standardized response mean (*SRM*) for the tests to show the responsiveness. *SRM* is calculated by dividing the mean change score by the standard deviation of change in scores [16]. The test with an *SRM* of 0.5 to 0.8 is considered to be moderately responsive, while tests with an *SRM* of 0.8 or larger are markedly responsive according to Cohen [17].

All methods were carried out in the accordance with the relevant guidelines and regulations in the manuscript.

## 3. Results

### 3.1. Demographic Characteristics

The basic information gathered from the 12,098 participants is presented in Table 1 based on the different age groups. Among all the participants, 7063 (58.4%) completed the test between 2013 and 2015, and most of them were from Shanghai, comprising 91.8% of the total. Children aged 13 months or older were the largest proportion, but the number of children aged from 1 to 5 months or 6 to 12 months was still large enough for analysis. The gender ratio was 1.19 males per female.

### 3.2. Reliability

Pearson correlation results, which are detailed in Table 2 and Table 3, showed that developmental age and chronological age were highly associated no matter where the subjects were from or when they came for the tests (*r* ranged from 0.89 to 0.98).

Children aged from 1 to 5 months cannot be assessed in the Self-Help domain. As detailed in Table 4, the Cronbach’s alpha coefficients are quite high (0.71 to 0.99) in six domains among all the subgroups. As presented in Table 5, standard errors of measurement are acceptable (0.15 to 2.76). However, the *SEM* for older children, such as the group aged 25 to 36 months is larger than that for younger children, such as the group aged from 13 to 24 months, and from 6 to 12 months versus 1 to 5 months.

### 3.3. Validity

Table 6 and Table 7, respectively, show the matrix of zero-order correlations and partial correlations in different provinces from 2013 to 2015, while those from 2016 to 2019 are presented in Table 8 and Table 9. The correlation of evaluation results in various areas can effectively reflect whether one dimension can be distinguished from the others. Before the correction of chronological age, the correlation coefficients between developmental age in different areas for children in the two periods were high (0.85–0.97). After controlling for chronological age, the correlation coefficient between the dimensions decreased markedly, ranging from 0.18 to 0.78. Among these partial correlation coefficients, the ones between fine movement and cognition, language and cognition were notably higher, reaching 0.62 and 0.63, respectively, in Shanghai, and 0.78 and 0.72 in other provinces.

### 3.4. Responsiveness

For children who completed the two tests with a time gap of 3 months (Table 10), paired *t*-test results showed that developmental age, obtained via the C-LAP system, changed significantly (*p* < 0.001) over time. *SRM* (0.74 to 2.45) also showed that the C-LAP system had excellent responsiveness to time changes, which reflects the real developmental improvement of children regardless of domain or age group. *SRM* in the younger age group of 1~12 months is larger than that in older age groups, indicating greater responsiveness.

For children who completed two tests with a time gap of 1 month or 2 months (Table 11), paired *t*-test results also showed that developmental age changed significantly (*p* < 0.001) between these two tests. *SRM* coefficients of the tests within 1 month were smaller than that within 2 months, illustrating the better responsiveness of the C-LAP system to a 2-month time change compared to a 1-month time change. *SRM* indicated a moderate responsiveness for the domains Fine Motor, Language, and Self-Help in the C-LAP system to tests with a time gap of 1 month. Excellent responsiveness was presented by the *SRM* of the other domains of the C-LAP system to a 1-month time change or all the domains to a 2-month time change.

## 4. Discussion

Previous studies have shown that E-LAP system has a high level of raters’ reliability, internal consistency, and convergent validity [8,18,19]. The retest consistency was also reported as excellent, ranging from 0.93 to 0.998. The E-LAP was indicated to be reliable and valid for assessing children’s development. For the C-LAP, this study found that, in the two time periods of 2013–2015 and 2016–2019, the correlation coefficients of chronological and measured developmental age of children in Shanghai and other provinces were between 0.89 and 0.97, indicating that the developmental age obtained via the C-LAP test could be reliably associated with the chronological age for children. Compared to the studies evaluating the reliability and validity of the E-LAP, this study demonstrated that the C-LAP had a relatively higher Cronbach’s alpha of 0.71 to 0.99, with standard errors of measurement lower than 3. These data indicated high internal consistency of the C-LAP. We found that *SEM* coefficients in older age groups (e.g., 25–36 months) are relatively higher than those in younger age group (e.g., 13–24 months), while the Cronbach’s alpha coefficients do not have much difference. Since *SEM* is positively related to the standard deviation of the observed scores and negatively related to Cronbach’s alpha, the standard deviation of the developmental scores in older age groups should be larger, which indicates that the C-LAP is more reliable when assessing younger children’s developmental age.

As developmental age in six domains was highly associated with chronological age, the developmental ages in each domain should be highly associated with each other if chronological age is not controlled, which is shown in the zero-order correlation results below the diagonal of the correlation matrix. The results of partial correlation, which is presented above the correlation matrix, showed that, after controlling for chronological age, the developmental ages of different dimensions of children in Shanghai were correlated to a certain extent, but none of these partial correlation coefficients were above 0.7; thus, the structural validity were demonstrated to be relatively ideal. However, in provinces other than Shanghai, the partial correlations between fine motor and cognition, language and cognition were higher (>0.7). This, in a sense, indicated that these dimensions were associated with each other, and other recent studies have also documented the obvious relationship between them [20]. However, there were several shared items in the C-LAP system, making the partial correlation coefficients between fine motor and cognition, as well as language and cognition, higher than others. Additionally, the relatively smaller sample size from other provinces when compared to Shanghai, and the direct combination of data from different provinces, may also cause these partial correlation coefficients to be larger than those in Shanghai. Hence, a larger sample size is needed for further verification in other provinces.

In this study, longitudinal data were also used to identify the difference in the developmental age in different domains, acquired from two assessments of the same research object with time gaps of from 1 to 3 months. The results indicated that the differences between the two measurements were significant three, two or even one month apart, which illustrated the high discriminant validity of C-LAP system, which sensitively detected the short-term changes in children’s neuropsychological development in different areas. The standardized response *mean (SRM)* also demonstrated that the C-LAP system was moderately or markedly responsive towards children’s development. We can also find that the C-LAP was more responsive in younger age groups compared with older age groups. This may be the result of the faster development of younger children compared to older ones. Thus, the developmental changes in younger children could be much easier to detect using the C-LAP system.

However, this study has several limitations. First, children with development disorders or diseases were not included in this study, so we could not analyze the reliability and validity of the C-LAP when assessing children with disabilities. Second, the test-retest reliability and concurrent validity were not analyzed due to the lack of corresponding data, which will be examined in a further study. Third, more than 90% of the participants were from Shanghai, which restricted this research. Even if we gathered 989 participants from other provinces in China with a high Cronbach’s coefficient, it would still be necessary to re-examine the reliability and validity of the C-LAP system.

## 5. Conclusions

This study aimed to evaluate the C-LAP system, which was translated from the E-LAP to Chinese, and found that the C-LAP is generally reliable, valid, and moderately to markedly responsive in Shanghai. The C-LAP was found to be more appropriate for younger children compared with older ones regarding its reliability and responsiveness. However, the results for older children, aged from 1 to 3 years, were still outstanding. Thus, the C-LAP is suitable for assessing child development in Shanghai, but still needs confirmation when used in other provinces in China due to the great differences between regions of China.

## Figures and Tables

**Table 1:** Distribution of period, province, gender, parents’ educational, parents’ age.

Variable	Participants’ Chronological Age (m)				
	Total (*n* = 12,098)	1~5 (*n* = 839)	6~12 (*n* = 2454)	13~24 (*n* = 4253)	25~36 (*n* = 4552)
Period, *n* (%)					
2013–2015	7063 (58.4)	418 (49.8)	971 (39.6)	2428 (57.1)	3246 (71.3)
2016–2019	5035 (41.6)	421 (50.2)	1483 (60.4)	1825 (42.9)	1306 (28.7)
Province, *n* (%)					
Shanghai	11,109 (91.8)	685 (81.6)	2266 (92.3)	3982 (93.6)	4176 (91.7)
Other	989 (8.2)	154 (18.4)	188 (7.7)	271 (6.4)	376 (8.3)
Gender, *n* (%)					
Male	6569 (54.3)	477 (56.9)	1346 (54.8)	2281 (53.6)	2465 (54.2)
Female	5529 (45.7)	362 (43.1)	1108 (45.2)	1972 (46.4)	2087 (45.8)
Father’s education, *n* (%)					
Postgraduate	917 (7.6)	110 (13.1)	267 (10.9)	303 (7.1)	237 (5.2)
Bachelor	6009 (49.7)	575 (68.5)	1496 (61)	2098 (49.3)	1840 (40.4)
Junior college	3563 (29.5)	112 (13.3)	484 (19.7)	1299 (30.5)	1668 (36.6)
High school or below	1609 (13.3)	42 (5)	207 (8.4)	553 (13)	807 (17.7)
Mather’s education, *n* (%)					
Postgraduate	695 (5.7)	76 (9.1)	222 (9)	233 (5.5)	164 (3.6)
Bachelor	6127 (50.6)	586 (69.8)	1534 (62.5)	2119 (49.8)	1888 (41.5)
Junior college	3634 (30)	132 (15.7)	484 (19.7)	1330 (31.3)	1688 (37.1)
High school or below	1642 (13.6)	45 (5.4)	214 (8.7)	571 (13.4)	812 (17.8)
Paternal age (y), *n* (%)					
≤25	458 (3.8)	34 (4.1)	114 (4.6)	149 (3.5)	161 (3.5)
26–30	4495 (37.2)	268 (31.9)	794 (32.4)	1620 (38.1)	1813 (39.8)
31–35	5349 (44.2)	389 (46.4)	1158 (47.2)	1831 (43.1)	1971 (43.3)
36–40	1372 (11.3)	111 (13.2)	303 (12.3)	487 (11.5)	471 (10.3)
≥41	424 (3.5)	37 (4.4)	85 (3.5)	166 (3.9)	136 (3)
Maternal age (y), *n* (%)					
≤25	769 (6.4)	51 (6.1)	173 (7)	257 (6)	288 (6.3)
26–30	5901 (48.8)	373 (44.5)	1105 (45)	2070 (48.7)	2353 (51.7)
31–35	4485 (37.1)	345 (41.1)	970 (39.5)	1565 (36.8)	1605 (35.3)
36–40	819 (6.8)	59 (7)	183 (7.5)	305 (7.2)	272 (6)
≥41	124 (1)	11 (1.3)	23 (0.9)	56 (1.3)	34 (0.7)

Characteristics of the samples used for examining *SRM* for children’s developmental age obtained from two tests with a time gap of 1–3 months are shown in Tables S1 and S2.

**Table 2:** Pearson correlation coefficients between developmental age and chronological age from 2013 to 2015, by province.

Province	Domain	*n*	*Mean* of Developmental Age	*sd* of Developmental Age	*r*
Shanghai	Gross Motor	1655	15.46	9.59	0.96
	Fine Motor	1791	16.76	9.94	0.96
	Cognitive	1769	16.93	9.97	0.97
	Language	6459	21.32	8.77	0.94
	Self-Help	5925	22.11	7.65	0.92
	Social Emotional	6217	22.06	9.57	0.92
Other Provinces	Gross Motor	591	18.09	10.04	0.91
	Fine Motor	641	19.15	10.56	0.92
	Cognitive	548	19.13	11.01	0.93
	Language	567	20.34	10.47	0.93
	Self-Help	462	20.43	8.14	0.89
	Social Emotional	562	19.66	11.09	0.92

**Table 3:** Pearson correlation coefficients between developmental age and chronological age from 2016 to 2019 by province.

Province	Domain	*n*	*Mean* of Developmental Age	*sd* of Developmental Age	*r*
Shanghai	Gross Motor	1878	11.75	7.77	0.97
	Fine Motor	1868	12.05	8.53	0.97
	Cognitive	1827	12.38	8.64	0.98
	Language	4720	16.12	8.86	0.96
	Self-Help	4319	17.34	8.17	0.96
	Social Emotional	4679	16.3	9.16	0.95
Other Provinces	Gross Motor	252	16.97	10.67	0.96
	Fine Motor	254	17.7	11.46	0.97
	Cognitive	250	18.27	11.88	0.97
	Language	253	18	11.61	0.96
	Self-Help	199	20.62	8.5	0.95
	Social Emotional	249	18.35	12.52	0.95

**Table 4:** Cronbach’s alpha coefficients of the C-LAP’s six domains by period and province.

Period	Province	Domain	Chronological Age (m)				Total
			1–5	6–12	13–24	25–36	
2013–2015	Shanghai	Gross Motor	0.93	0.96	0.94	0.88	0.98
		Fine Motor	0.88	0.94	0.93	0.91	0.99
		Cognitive	0.89	0.95	0.95	0.95	0.99
		Language	0.78	0.88	0.93	0.91	0.98
		Self-Help	NA	0.84	0.95	0.86	0.97
		Social Emotional	0.73	0.86	0.84	0.76	0.95
	Other provinces	Gross Motor	0.98	0.97	0.97	0.77	0.99
		Fine Motor	0.99	0.95	0.96	0.86	0.99
		Cognitive	0.98	0.96	0.97	0.94	0.99
		Language	0.97	0.89	0.95	0.92	0.98
		Self-Help	NA	0.94	0.96	0.89	0.98
		Social Emotional	0.82	0.9	0.91	0.75	0.97
2016–2019	Shanghai	Gross Motor	0.91	0.96	0.91	0.75	0.98
		Fine Motor	0.87	0.93	0.92	0.89	0.98
		Cognitive	0.87	0.95	0.94	0.94	0.99
		Language	0.78	0.87	0.92	0.92	0.98
		Self-Help	NA	0.84	0.94	0.88	0.98
		Social Emotional	0.76	0.84	0.82	0.79	0.95
	Other provinces	Gross Motor	0.98	0.97	0.94	0.71	0.99
		Fine Motor	0.91	0.95	0.94	0.89	0.99
		Cognitive	0.93	0.96	0.95	0.93	0.99
		Language	0.87	0.94	0.94	0.91	0.99
		Self-Help	NA	0.9	0.95	0.84	0.98
		Social Emotional	0.86	0.91	0.82	0.81	0.98

**Table 5:** Standard errors of measurement of the C-LAP’s six domains by period and province.

Period	Province	Domain	Chronological Age (m)				Total
			1–5	6–12	13–24	25–36	
2013–2015	Shanghai	Gross Motor	0.44	0.48	0.91	1.45	0.95
		Fine Motor	0.48	0.59	1.14	1.43	1.12
		Cognitive	0.5	0.53	0.93	1.09	0.99
		Language	0.77	0.88	0.99	1.42	1.39
		Self-Help	NA	0.92	0.8	1.86	1.3
		Social Emotional	0.98	0.94	1.53	2.7	2.06
	Other provinces	Gross Motor	0.25	0.43	0.6	2.35	0.82
		Fine Motor	0.15	0.51	0.86	1.82	1.05
		Cognitive	0.2	0.45	0.77	1.17	0.96
		Language	0.3	0.86	0.82	1.4	1.19
		Self-Help	NA	0.56	0.69	1.61	1.19
		Social Emotional	0.8	0.79	1.12	2.76	1.71
2015–2019	Shanghai	Gross Motor	0.48	0.5	1.07	2.42	1.09
		Fine Motor	0.5	0.62	1.26	1.64	1.23
		Cognitive	0.54	0.54	1	1.11	1.08
		Language	0.76	0.94	1.09	1.33	1.37
		Self-Help	NA	0.93	0.83	1.72	1.19
		Social Emotional	0.93	1.03	1.6	2.52	2.05
	Other provinces	Gross Motor	0.23	0.46	0.87	2.21	0.75
		Fine Motor	0.41	0.56	1.08	1.62	0.92
		Cognitive	0.39	0.48	0.91	1.2	0.8
		Language	0.59	0.61	0.92	1.44	1.03
		Self-Help	NA	0.72	0.8	1.97	1.13
		Social Emotional	0.71	0.75	1.63	2.41	1.42

**Table 6:** Zero-order correlations and partial correlations controlled for age among the C-LAP’s six domains among participants from Shanghai between 2013 and 2015.

	Gross Motor	Fine Motor	Cognitive	Language	Self-Help	Social Emotional
Gross Motor	1.00	0.29	0.32	0.35	0.32	0.26
Fine Motor	0.95	1.00	0.62	0.48	0.44	0.38
Cognitive	0.95	0.97	1.00	0.63	0.43	0.43
Language	0.95	0.96	0.97	1.00	0.37	0.34
Self-Help	0.92	0.94	0.94	0.91	1.00	0.29
Social Emotional	0.93	0.94	0.95	0.91	0.88	1.00

Partial correlation coefficients are presented above the diagonal while zero-order correlation coefficients are presented below the diagonal.

**Table 7:** Zero-order correlations and partial correlations, controlled for age, among the C-LAP’s six domains among participants from other provinces between 2013 and 2015.

	Gross Motor	Fine Motor	Cognitive	Language	Self-Help	Social Emotional
Gross Motor	1.00	0.44	0.40	0.26	0.31	0.18
Fine Motor	0.92	1.00	0.76	0.44	0.47	0.33
Cognitive	0.91	0.97	1.00	0.62	0.45	0.38
Language	0.89	0.92	0.95	1.00	0.25	0.37
Self-Help	0.85	0.90	0.91	0.86	1.00	0.30
Social Emotional	0.88	0.91	0.92	0.91	0.87	1.00

Partial correlation coefficients are presented above the diagonal while zero-order correlation coefficients are presented below the diagonal.

**Table 8:** Zero-order correlations and partial correlations, controlled for age, among the C-LAP’s six domains among participants from Shanghai between 2016 and 2019.

	Gross Motor	Fine Motor	Cognitive	Language	Self-Help	Social Emotional
Gross Motor	1.00	0.39	0.27	0.28	0.26	0.31
Fine Motor	0.95	1.00	0.62	0.40	0.31	0.39
Cognitive	0.95	0.97	1.00	0.54	0.38	0.41
Language	0.95	0.96	0.97	1.00	0.39	0.41
Self-Help	0.92	0.94	0.94	0.91	1.00	0.38
Social Emotional	0.93	0.94	0.95	0.91	0.88	1.00

Partial correlation coefficients are presented above the diagonal, while zero-order correlation coefficients are presented below the diagonal.

**Table 9:** Zero-order correlations and partial correlations, controlled for age, among the C-LAP’s six domains among participants from other provinces between 2016 and 2019.

	Gross Motor	Fine Motor	Cognitive	Language	Self-Help	Social Emotional
Gross Motor	1.00	0.50	0.60	0.50	0.33	0.46
Fine Motor	0.92	1.00	0.78	0.57	0.35	0.46
Cognitive	0.91	0.97	1.00	0.72	0.48	0.60
Language	0.89	0.92	0.95	1.00	0.43	0.58
Self-Help	0.85	0.91	0.91	0.86	1.00	0.34
Social Emotional	0.88	0.90	0.92	0.91	0.87	1.00

Partial correlation coefficients are presented above the diagonal, while zero-order correlation coefficients are presented below the diagonal.

**Table 10:** Paired *t*-test result and standardized response mean (*SRM*) for children’s developmental age, obtained from two tests with a time gap of 3 months.

Age Group (m)	Domain	*Mean* of Difference	*std* of Difference	*SRM*	*t*
1~12	Gross Motor	2.99	1.22	2.45	32.65 ^***^
1~12	Fine Motor	2.93	1.24	2.37	31.61 ^***^
1~12	Cognitive	2.79	1.21	2.31	30.65 ^***^
1~12	Language	3.33	1.35	2.46	32.56 ^***^
1~12	Self-Help	2.69	1.26	2.14	24.15 ^***^
1~12	Social Emotional	3.09	1.43	2.16	28.63 ^***^
13~24	Gross Motor	3.15	1.61	1.96	27.52 ^***^
13~24	Fine Motor	3.29	2.03	1.62	22.79 ^***^
13~24	Cognitive	3.35	1.79	1.87	26.21 ^***^
13~24	Language	3.66	2.63	1.39	19.35 ^***^
13~24	Self-Help	2.89	1.69	1.71	23.83 ^***^
13~24	Social Emotional	3.43	1.98	1.73	24.32 ^***^
25~36	Gross Motor	2.57	3.49	0.74	5.25 ^***^
25~36	Fine Motor	3.00	3.13	0.96	6.64 ^***^
25~36	Cognitive	3.11	2.41	1.29	8.85 ^***^
25~36	Language	3.67	3.51	1.04	7.23 ^***^
25~36	Self-Help	3.14	2.74	1.15	8.03 ^***^
25~36	Social Emotional	4.26	3.55	1.20	7.87 ^***^

*** *p* value < 0.001.

**Table 11:** Paired *t-*test result and standardized response mean (*SRM*) for children’s developmental age obtained from two tests with a time gap of 1–2 months.

Time Gap (m)	Domain	*n*	*Mean* of Difference	*std* of Difference	*SRM*	*t*
1	Gross Motor	120	1.62	2.14	0.76	7.61 ^***^
	Fine Motor	120	1.65	2.37	0.70	7.00 ^***^
	Cognitive	120	2.29	2.21	1.04	10.16 ^***^
	Language	120	2.60	3.92	0.66	6.80 ^***^
	Self-Help	120	2.35	3.50	0.67	6.80 ^***^
	Social Emotional	120	1.78	2.00	0.89	9.02 ^***^
2	Gross Motor	200	2.56	1.95	1.31	18.32 ^***^
	Fine Motor	200	2.59	1.90	1.36	18.88 ^***^
	Cognitive	200	2.62	1.88	1.40	18.26 ^***^
	Language	200	2.80	2.46	1.14	15.85 ^***^
	Self-Help	200	2.49	2.07	1.20	16.55 ^***^
	Social Emotional	200	2.33	1.93	1.20	16.12 ^***^

*** *p* value < 0.001.

## Data Availability

The datasets are available on reasonable request. If anyone wants to get access to the data, please send an email to chend19@fudan.edu.cn.

## Supplementary Materials

The following are available online at https://www.mdpi.com/article/10.3390/children8110974/s1, Table S1: Characteristics of the samples used for examining SRM for children’s developmental age obtained from two tests with a time gap of 3 months, Table S2: Characteristics of the samples used for examining SRM for children’s developmental age obtained from two tests with a time gap of 1–2 months.
