# Supplementary material for: Reliability, Validity, and Responsiveness of the Chinese Learning Accomplishment Profile (C-LAP)
Source: Children (Basel). 2021 Oct 28;8(11):974. doi: 10.3390/children8110974 (PMC8622609; doi:10.3390/children8110974)
Supplement: Supplementary file 1 [file children-08-00974-s001.zip › children-1384902-supplementary.pdf]

Table S1. Characteristics of the samples used for examining *SRM* for children's developmental age obtained from two tests with a time gap of 3 months.

**Variable**	**Total**	**Participants' Chronological Age (m)**			
		**1~5**	**6~12**	**13~24**	**25~36**
	**(*n* = 200)**	**(*n* = 22)**	**(*n* = 62)**	**(*n* = 84)**	**(*n* = 32)**
Period, n (%)					
2013–2015	79 (39.5)	7 (31.8)	31 (50)	32 (38.1)	9 (28.1)
2016–2019	121 (60.5)	15 (68.2)	31 (50)	52 (61.9)	23 (71.9)
Province, n (%)					
Shanghai	177 (88.5)	22 (100)	53 (85.5)	73 (86.9)	29 (90.6)
Other	23 (11.5)	0 (0)	9 (14.5)	11 (13.1)	3 (9.4)
Gender, n (%)					
Male	106 (53)	10 (45.5)	34 (54.8)	45 (53.6)	17 (53.1)
Female	94 (47)	12 (54.5)	28 (45.2)	39 (46.4)	15 (46.9)
Father's education, n (%)					
Postgraduate	24 (12)	2 (9.1)	8 (12.9)	10 (11.9)	4 (12.5)
Bachelor	150 (75)	18 (81.8)	45 (72.6)	62 (73.8)	25 (78.1)
Junior college	23 (11.5)	1 (4.5)	8 (12.9)	11 (13.1)	3 (9.4)
High school or below	3 (1.5)	1 (4.5)	1 (1.6)	1 (1.2)	0 (0)
Mather's education, n (%)					
Postgraduate	15 (7.5)	1 (4.5)	6 (9.7)	6 (7.1)	2 (6.2)
Bachelor	159 (79.5)	18 (81.8)	50 (80.6)	66 (78.6)	25 (78.1)
Junior college	25 (12.5)	2 (9.1)	6 (9.7)	12 (14.3)	5 (15.6)
High school or below	1 (0.5)	1 (4.5)	0 (0)	0 (0)	0 (0)
Paternal age (y), n (%)					
≤25	6 (3)	0 (0)	3 (4.8)	3 (3.6)	0 (0)
26–30	53 (26.5)	9 (40.9)	17 (27.4)	19 (22.6)	8 (25)
31–35	92 (46)	10 (45.5)	29 (46.8)	39 (46.4)	14 (43.8)
36–40	40 (20)	2 (9.1)	11 (17.7)	18 (21.4)	9 (28.1)
≥41	9 (4.5)	1 (4.5)	2 (3.2)	5 (6)	1 (3.1)
Maternal age (y), n (%)					
≤25	9 (4.5)	2 (9.1)	3 (4.8)	3 (3.6)	1 (3.1)
26–30	84 (42)	7 (31.8)	29 (46.8)	34 (40.5)	14 (43.8)
31–35	85 (42.5)	11 (50)	23 (37.1)	38 (45.2)	13 (40.6)
36–40	18 (9)	2 (9.1)	6 (9.7)	7 (8.3)	3 (9.4)
≥41	4 (2)	0 (0)	1 (1.6)	2 (2.4)	1 (3.1)

Table S2. Characteristics of the samples used for examining *SRM* for children's developmental age obtained from two tests with a time gap of 1–2 months.

Variable	Total	Participants' Chronological Age (m)			
		1~5	6~12	13~24	25~36
	(n = 120)	(n = 4)	(n = 19)	(n = 47)	(n = 50)
Period, n (%)					
2013–2015	77 (64.2)	3 (75)	8 (42.1)	30 (63.8)	36 (72)
2016–2019	43 (35.8)	1 (25)	11 (57.9)	17 (36.2)	14 (28)
Province, n (%)					
Shanghai	102 (85)	2 (50)	18 (94.7)	41 (87.2)	41 (82)
Other	18 (15)	2 (50)	1 (5.3)	6 (12.8)	9 (18)
Gender, n (%)					
Male	77 (64.2)	3 (75)	12 (63.2)	27 (57.4)	35 (70)
Female	43 (35.8)	1 (25)	7 (36.8)	20 (42.6)	15 (30)
Father's education, n (%)					
Postgraduate	8 (6.7)	0 (0)	2 (10.5)	3 (6.4)	3 (6)
Bachelor	83 (69.2)	3 (75)	13 (68.4)	32 (68.1)	35 (70)
Junior college	22 (18.3)	0 (0)	3 (15.8)	9 (19.1)	10 (20)
High school or below	7 (5.8)	1 (25)	1 (5.3)	3 (6.4)	2 (4)
Mather's education, n (%)					
Postgraduate	8 (6.7)	0 (0)	2 (10.5)	4 (8.5)	2 (4)
Bachelor	85 (70.8)	3 (75)	13 (68.4)	34 (72.3)	35 (70)
Junior college	19 (15.8)	0 (0)	4 (21.1)	6 (12.8)	9 (18)
High school or below	8 (6.7)	1 (25)	0 (0)	3 (6.4)	4 (8)
Paternal age (y), n (%)					
≤25	4 (3.3)	0 (0)	0 (0)	4 (8.5)	0 (0)
26–30	62 (51.7)	2 (50)	8 (42.1)	23 (48.9)	29 (58)
31–35	42 (35)	2 (50)	8 (42.1)	16 (34)	16 (32)
36–40	6 (5)	0 (0)	1 (5.3)	3 (6.4)	2 (4)
≥41	6 (5)	0 (0)	2 (10.5)	1 (2.1)	3 (6)
Maternal age (y), n (%)					
≤25	6 (5)	0 (0)	0 (0)	5 (10.6)	1 (2)
26–30	77 (64.2)	3 (75)	13 (68.4)	29 (61.7)	32 (64)
31–35	28 (23.3)	1 (25)	4 (21.1)	11 (23.4)	12 (24)
36–40	7 (5.8)	0 (0)	2 (10.5)	1 (2.1)	4 (8)
≥41	2 (1.7)	0 (0)	0 (0)	1 (2.1)	1 (2)
